# In Vivo Accelerator-Based Boron Neutron Capture Therapy for Spontaneous Tumors in Large Animals: Case Series

**DOI:** 10.3390/biology11010138

**Published:** 2022-01-14

**Authors:** Vladimir Kanygin, Aleksandr Kichigin, Alexander Zaboronok, Anna Kasatova, Elena Petrova, Alphiya Tsygankova, Evgenii Zavjalov, Bryan J. Mathis, Sergey Taskaev

**Affiliations:** 1 Laboratory of Medical and Biological Problems of BNCT, Department of Physics, Novosibirsk State University, 1 Pirogov Str., 630090 Novosibirsk, Russia; kanigin@mail.ru (V.K.); sam@211.ru (A.K.); alphiya@yandex.ru (A.T.); zavjalov@bionet.nsc.ru (E.Z.); 2 Department of Neurosurgery, Faculty of Medicine, University of Tsukuba, 1-1-1 Tennodai, Tsukuba 305-8575, Ibaraki, Japan; 3 Budker Institute of Nuclear Physics, Siberian Branch of Russian Academy of Sciences, 11, Acad. Lavrentieva Ave., 630090 Novosibirsk, Russia; yarullinaai@yahoo.com (A.K.); taskaev@inp.nsk.su (S.T.); 4 Veterinary Clinic “Best”, 57 Frunze Str., 630005 Novosibirsk, Russia; vet.best@mail.ru; 5 Nikolaev Institute of Inorganic Chemistry SB RAS, 3, Acad. Lavrentieva Ave., 630090 Novosibirsk, Russia; 6 Center for Genetic Resources of Laboratory Animals, Institute of Cytology and Genetics SB RAS, 10, Acad. Lavrentieva Ave., 630090 Novosibirsk, Russia; 7 International Medical Center, University of Tsukuba Hospital, 2-1-1 Amakubo, Tsukuba 305-8576, Ibaraki, Japan; bmathis@md.tsukuba.ac.jp; 8 Laboratory of BNCT, Department of Physics, Novosibirsk State University, 1 Pirogov Str., 630090 Novosibirsk, Russia

**Keywords:** boron neutron capture therapy, accelerator-based neutron source, malignant tumors, veterinary medicine

## Abstract

**Simple Summary:**

Accelerator-based neutron sources for boron neutron capture therapy (BNCT) are potentially more accessible than nuclear reactors but many technical issues in clinical trials and further routine therapy remain to be studied. We aim to broaden the understanding of these issues with a study of BNCT in 10 cats and dogs, highlighting practical issues, using an accelerator-based neutron source. Using larger animals with tumors mimicking human clinical progression is an important intermediate step to clinical BNCT development.

**Abstract:**

(1) Background: accelerator-based neutron sources are a new frontier for BNCT but many technical issues remain. We aimed to study such issues and results in larger-animal BNCT (cats and dogs) with naturally occurring, malignant tumors in different locations as an intermediate step in translating current research into clinical practice. (2) Methods: 10 pet cats and dogs with incurable, malignant tumors that had no treatment alternatives were included in this study. A tandem accelerator with vacuum insulation was used as a neutron source. As a boron-containing agent, ^10^B-enriched sodium borocaptate (BSH) was used at a dose of 100 mg/kg. Animal condition as well as tumor progression/regression were monitored. (3) Results: regression of tumors in response to treatment, improvements in the overall clinical picture, and an increase in the estimated duration and quality of life were observed. Treatment-related toxicity was mild and reversible. (4) Conclusions: our study contributes to preparations for human BNCT clinical trials and suggests utility for veterinary oncology.

## 1. Introduction

Boron-neutron capture therapy (BNCT) is a cancer treatment technique based on the selective accumulation of ^10^B isotope-enriched compounds in the tumor followed by irradiation with a beam of thermal and epithermal neutrons [1]. The interaction between ^10^B and neutrons generates high-energy particles (alpha particles and lithium nuclei) that travel only a very short distance (5–9 μm, about the diameter of a mammalian cell) and inflicts lethal damage that leads to tumor cell death. Thus, by ensuring the selective accumulation of ^10^B in tumor cells followed by its irradiation with a flux of neutrons, a lethal damage cascade in tumor cells occurs while normal cells remain intact [1].

L-p-borphenylalanine (BPA) and sodium borocaptate (BSH) enriched with 99.9% ^10^B are well-studied boron-containing compounds [2]. BPA is actively taken up by tumor cells with the help of the amino acid L-transport system, against a background of increased proliferation and protein synthesis by tumor cells, while BSH accumulation in tumor tissue is associated with an angiogenic, enriched blood supply. However, the requirement to selectively accumulate only in the tumor render BPA and BSH suboptimal (as both also infiltrate normal cells) and research on the creation of tumor-selective, boron-containing compounds based on liposomes, nanoparticles and nanotubes, phages, and tumor-specific ligands of antibodies, etc. continues [3]. However, until these novel delivery technologies are refined, efforts should be directed towards improving the selectivity and concentration of BPA and BSH drugs in tumors [4].

Neutrons for BNCT may be sourced from both nuclear reactors and charged particle accelerators, with the latter being currently developed for safety reasons. Clinical studies conducted at nuclear reactors have shown effectiveness for malignant tumors in humans [5,6]. Compared to clinical trials conducted at the beginning of the BNCT era [7,8,9,10,11], recent studies employing other adjuvant modalities and technologically more advanced surgical methods, as well as the introduction of BPA and newer, more efficient drug delivery protocols and irradiation regimens, have demonstrated efficacy for diverse cancers [5,6]. Such cancers include newly diagnosed and recurrent malignant gliomas [12,13,14,15,16,17,18,19,20], recurrent malignant meningiomas [19,20,21,22,23], malignant melanomas [24,25,26,27,28], head and neck cancers (including squamous and non-squamous cell carcinoma) [29,30,31,32,33,34,35,36], liver cancer [37,38] and metastases [39], lung cancer [40] and metastases [41], and other malignances [42,43,44,45,46,47,48]. However, geopolitically, BNCT based on reactors has effectively ceased to exist, except in Japan, Taiwan, Argentina, and China. In Japan, clinical applications of reactor-based BNCT remain discontinued; however, preclinical and fundamental experiments with new boron compounds are still conducted at the Kyoto University Research Reactor Institute (KURRI) [49]. Meanwhile, in Taiwan and Argentina, the THOR and RA-6 reactors, respectively, are still clinically operational [50,51,52] and, in China, the 30 kW, reactor-based In-Hospital Neutron Irradiator-1 (IHNI-1) has been recently developed [53]. Nevertheless, BNCT globally depends on further development of accelerator-based neutron sources. In Japan, promising results of accelerator-based BNCT (AB-BNCT) clinical trials during the last several years [54,55] led to both the approval of an insurance-covered, standard clinical application in patients with head and neck cancer using a BNCT30 accelerator (Sumitomo Heavy Industries, Inc., Tokyo, Japan) and the approval of a world-first, BPA-based boron drug (Steboronine^®^) by Stella Pharma, Co. Ltd. (Tokyo, Japan) [56,57]. Several more AB-BNCT projects are ongoing in Japan, including accelerators developed by the University of Tsukuba (together with KEK and Mitsubishi Heavy Industries) [58,59], at the National Cancer Center in Tokyo [60], at the Nagoya University [61]. In Finland, a BNCT center is now online and ready for clinical application in Helsinki with an accelerator constructed by Neutron Therapeutics, Inc. (Danvers, MA, USA) [62,63]. TAE Life Sciences, LLC (Foothill Ranch, Lake Forest, CA, USA), based on the prototype constructed at the Budker Institute in Novosibirsk, Russia, has built its own accelerator-based neutron source (Alphabeam^TM^) and reported its installation at the Xiamen Humanity Hospital in China [64,65]. Meanwhile, the Chinese Academy of Sciences has reported on building their first accelerator-based facility for BNCT experiments at the Institute of High Energy Physics (Dongguan, Guangdong Province, China) using the China Spallation Neutron Source [66,67].

In Russia, a governmental program has been launched to boost the accelerator constructed at the Institute of Nuclear Physics in Novosibirsk into the clinical phase over the next few years [5,68]. Thus, before treating malignant tumors in humans using this accelerator-based neutron source, both physical and radiobiological studies of the beam must be performed to assess compliance with therapeutic and biosafety requirements. For this, in vitro studies on tumor cell cultures [69,70,71,72,73] and in vivo animal tumor models [74,75] play a key role. However, it should be recognized that in vitro and small animal tumor models (especially murine) cannot reproduce the full complexity of spontaneous tumor organization as well as humoral and microenvironmental factors. In addition, body size differences between humans and small laboratory animals often makes these models unable to accurately predict clinical efficacy as well as reliably detect undesirable adverse reactions.

On the other hand, treating spontaneous tumors in larger pets, mainly dogs and cats, allows for complex dosimetric parameters relevant to human radiation therapy. Second, dogs and cats tend to develop similar cancers in the same areas as humans [76]. Third, such pets live in similar environments as humans but the accelerated progress of animal cancers allow for rapid testing and results. A further argument is that the biological and treatment responses to tumors in pets are better models of human tissue reactions than tiny rodent bodies [77]. Thus, when feasible, these kinds of studies are a key intermediate step for testing BNCT technologies for clinical development.

Moreover, since veterinary BNCT itself requires similar, incremental research development [74], BNCT treatment of pets with spontaneous tumors is useful for development in clinical and veterinary oncology [52,78].

Since primary interest in BNCT is focused on head and neck cancers and infiltrative tumors, the malignancies considered suitable for testing in larger pets (cats/dogs) are those such as oral melanomas, oral squamous cell cancers, lung cancers, etc. The aim of this study is therefore to optimize therapeutic BNCT efficacy in the treatment of malignant tumors, as well as study the role of BNCT in veterinary medicine and delineate technical aspects for future clinical studies in humans using accelerator-based neutron sources.

## 2. Materials and Methods

In each case, informed consent from owners was secured. Additionally, the treatment protocols were approved by the interinstitutional commission on biological ethics at the Institute of Cytology and Genetics of the Siberian Branch of the Russian Academy of Sciences. In this case, 10 feline or canine patients with diverse malignant tumors with no alternative treatment options were included according to these inclusion criteria:

– Tumors could not be surgically removed due to high risks of postoperative complications, tumor after recurrence, or with a high likelihood of recurrence;
– A life and quality-of-life expectancy of 3 or more months for follow-up purposes;
– A somatic status adequate to withstand prolonged (2 h) anesthesia during irradiation;
– If the animal has previously received surgical treatment, there should be at least 3 weeks between the end of that treatment and BNCT.

Animal data are shown in Table 1.

Computed tomography (CT) or magnetic resonance imaging (MRI) were performed prior to treatment to determine the extent of spread, tumor size, and/or presence of distant metastases. Tomography method data were also used for treatment planning.

Epithermal neutron irradiation was carried out at the Budker Institute of Nuclear Physics in Novosibirsk, Russian Federation using the Vacuum Insulated Tandem Accelerator (VITA) neutron source with a lithium neutron-producing target [79,80,81,82]. The neutron-beam shaping assembly and detailed characteristics of the beam, including the neutron spectra, fast neutron, and photon components are described in previous reports [83,84,85,86,87]. Based on previous experimental data and spatial distribution of the beam components [88], plus an NMC code developed at IBRAE RAS [89] to simulate particle transport by the Monte Carlo method, we calculated neutron spectra and absorbed-dose depth distribution in simulated tumor, skin, and surrounding tissues prior to BNCT sessions. The dose rates (irradiation intensity) for each proton beam energy are provided in Table 2.

The required radiation dose was calculated from an assumed average boron concentration of 30 ppm in the tumor at the time of irradiation and 10 ppm in the surrounding tissues to achieve an estimated 30 Gy-eq in the tumor as a minimum calculated dose value during a single irradiation session. The skin was considered as a dose-limiting organ and the 18 Gy-eq upper dose limits for the skin were met [26]. In cases with repeated BNCT, for the second irradiation session, we took into account the previous dose if the same area was targeted and the subsequent dose was set to be lower than the first one. In some cases, if repeated irradiation was associated with tumor metastasis and involvement of another area, we conducted a full-dose irradiation.

Approximately one hour before irradiation, intravenous infusion of BSH solution at a dosage of 100 mg per kg of body weight in 0.9% NaCl at a total volume of 20 mL/kg of body weight was started for 1 h. After BSH infusion, animals were anesthetized intravenously using Dexdomitor (dexmedetomidine) at a dose of 20 mg/kg body weight and Zoletil (tiletamine and zolazepam) 100 at a dose of 3 mg/kg for large animals and 5 mg/kg for cats. Anesthesia was applied for the entire irradiation period, which lasted for approximately 2 h. After anesthesia induction (within several minutes after the BSH infusion was finished), a blood sample (1 mL) was taken from the peripheral vein. After blood sampling, the animal was carefully positioned under the target of the accelerator-based neutron source on a height-adjustable table, which took ≤10 min. The irradiation zone was located no more than 1 cm from the accelerator-based neutron source target and a laser was used to center on skin markings. The animals were placed in a physiological position that ensured adequate breathing, using rollers and soft cushions, and placed for maximum contact with hard surfaces, with fixation of limbs and torso via soft straps attached directly to the table. If the tumor was large and diffuse, more than one irradiation field (in front and from the opposite side, behind the tumor) was used. After irradiation, Antisedan (atipamezole hydrochloride) at 10 mg/kg body weight for dogs and 5 mg/kg for cats was used for awakening. The second blood sample was taken within ≤10 min after irradiation was finished.

Both blood samples collected after boron infusion and after the radiation session were analyzed by inductively coupled plasma atomic emission spectrometry (ICP-AES, ICPE-9800, Shimadzu Co., Ltd., Kyoto, Japan). No tumor boron concentrations were measured (since one aim was to predict tumor concentrations) but blood boron concentrations ranged from 60 to 167 ppm before irradiation and from 32 to 119 ppm after irradiation. To predict boron concentrations in tumors, tumor/blood ratios of 1:1 and tumor/healthy tissue ratios of 3:1 were adopted and used [52].

Data on boron concentration in blood and exposure parameters are shown in Table 3.

After BNCT, the somatic condition of the animals was monitored weekly for 3 to 6 months. Animal appetite, activity on a five-point scale, and body weight were assessed. Tumor response to treatment was assessed on the basis of CT scans performed 1 to 3 months after BNCT. Post-radiation reactions on the skin and coats of the animals were recorded.

## 3. Results

Presented for each of the animals as individual clinical cases:

### 3.1. Case 1

**Daya**—a dog, female, 12 years old.

*Diagnosis*: Soft tissue tumor of the facial skull with nasal septum displacement and nasopharyngeal obstruction.

*Complaints, anamnesis*: Impaired nasal breathing and nasal discharge.

*Examination findings:* Head CT: complete absence of nasal cavities on the left side, nasal septum displaced to the right. Osteodestruction of the nasal bone and orbit of the eye on the left side. Complete obstruction of nasopharynx with soft tissue component. Lysis of the frontal bone.

*Surgical treatment:* Dorsal rhinotomy for nasal breathing relief a month before BNCT with collection of material for histological study.

*Histological report:* Osteosarcoma of the nasal cavity.

*Post radiation monitoring and dynamics:* General condition was satisfactory during post radiation monitoring and improvement of nasal breathing was observed. Visual decreases in the visible part of the tumor occurred.

After 3.5 months, a contrast-CT scan of the head was performed; no negative dynamics were found, there was no soft tissue component, the mucosa was moderately hyperplasticized in the area of osteolysis, and the lymph nodes were not enlarged. However, CT of the chest cavity with contrast in the right lung lobes revealed the presence of 4 metastatic foci in the lung tissue. Taking into account the absence of the option of their surgical removal, a decision was made to repeat lung BNCT. At 87 days after the first head tumor BNCT session, the second BNCT session for lung metastases was carried out. One month after the second BNCT session, general condition was satisfactory and the owners did not complain about decreases in the animal’s activity. Five courses of carboplatin chemotherapy were performed. 

**Figure 1:**
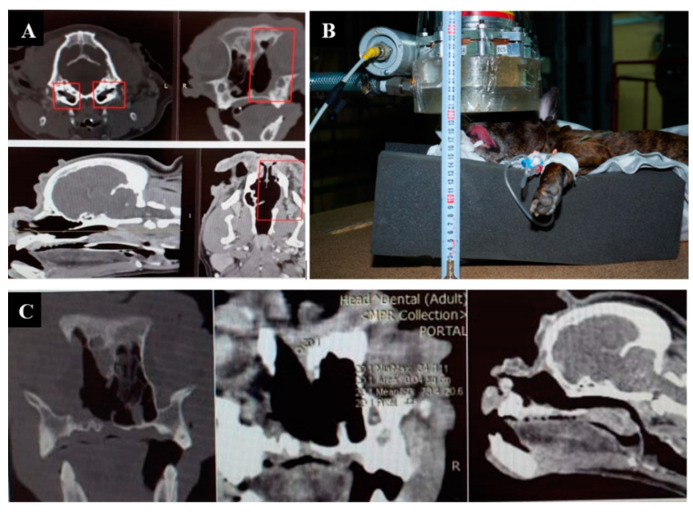
A dog named “Daya”. (**A**)—CT of the head before irradiation. (**B**)—positioning of the animal during the irradiation session. (**C**)—CT of the head 3 months after irradiation.

The imaging, positioning, and irradiation settings are shown in Figure 1.

Daya is alive as of 7 January 2022, with no signs of negative dynamics. According to a CT scan of the lungs, the metastases have decreased in size. No tumor tissue is visible in the nasal area.

### 3.2. Case 2

**Perchik**—a male cat, 4 years old.

*Diagnosis*: Volumetric mass of the frontal bone and volumetric mass of the mammary gland.

*Complaints, anamnesis*: Presence of volumetric masses with signs of dynamic growth. Start of chemotherapy by doxorubicin was accompanied by the animal’s refusal to eat, due to which the treatment was stopped.

*Examination findings:* Head CT—osteolytic focal lesion of the frontal bone on the left side with involvement of the left frontal sinus. Volumetric mass of the mammary gland of the 3rd lobe on the right with a solid structure up to 0.8 mm and inguinal lymph nodes were moderately enlarged up to 1.1 cm. No metastatic lesions of the thoracic or abdominal cavity were revealed.

*Histological report*: Carcinoma.

*Post irradiation follow-up and dynamics:* Purulent aseptic pleuritis was diagnosed 2 days after BNCT, apparently related to immunosuppression. Pleurocentesis was carried out, followed by a course of antibiotic therapy, after which the condition stabilized. Feeding was compulsory for one month. One month after BNCT there was a twofold reduction in tumor volume, the condition of the animal remained satisfactory, and the size of the mammary gland tumor in dynamics did not change. Two months after BNCT a repeated CT scan was performed and growth of the mass was observed. 

**Figure 2:**
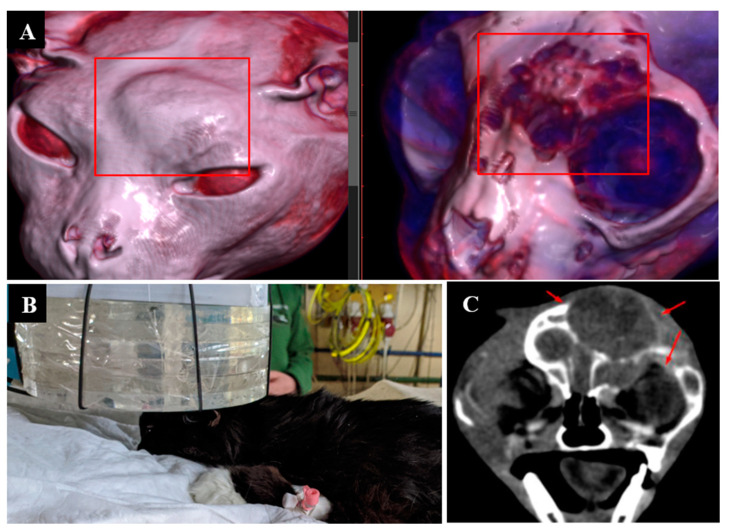
A cat named “Perchik”. (**A**)—CT of the head, chest and abdomen before irradiation. (**B**)—positioning of the animal during the irradiation session. (**C**)—CT of the head 2 months after irradiation.

Irradiation imaging and positioning are shown in Figure 2.

### 3.3. Case 3

**Kira**—a female cat, six years old.

*Diagnosis:* Soft tissue tumor of the back.

*Complaints, medical history:* Removal of a large mass (fibrosarcoma) of the soft tissues of the back. The tumor recurred 2 months after the operation.

CT scan of the chest and abdomen with contrast enhancement revealed a solid mass of subcutaneous tissue in the area of 7–8 spinous processes on the left side, connected with the underlying tissues (muscles). Regional lymph nodes were not changed and there were no focal masses in the thoracic and abdominal cavity.

*Tumor size before irradiation:* 1.5 × 2.1 × 1.0 cm.

*Histological report:* Fibrosarcoma.

*Post-radiation follow-up and dynamics:* Repeated contrast-CT scan of the thoracic and abdominal cavity detecting a volumetric mass of the same size was performed 3 weeks later. A single mass, located in the upper back near the neck with adjacent spinous processes, was removed one month after the first BNCT session. The animal was discharged in satisfactory condition and, 5 weeks after surgical treatment, a second session of BNCT was performed on the postoperative area. The state of the animal was satisfactory one week after the repeated session of BNCT and the owners have not complained of any decrease in the activity of the animal.

**Figure 3:**
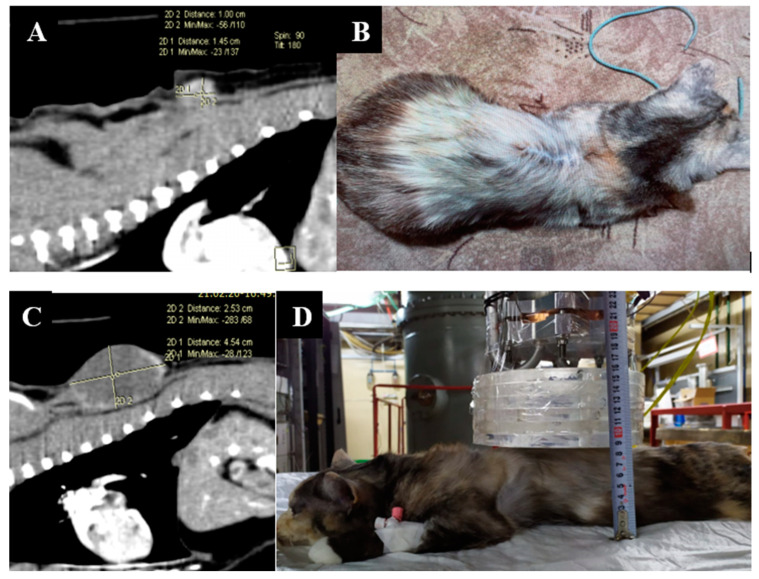
A cat named “Kira”. (**A**)—CT scan of the chest cavity, tumor of the soft tissues of the back before surgical treatment. (**B**)—condition after surgical treatment. (**C**)—Recurrent tumor of the soft tissues of the back. (**D**)—positioning of the animal during the repeated irradiation session.

Animal appearance, irradiation positioning, and imaging results are shown in Figure 3.

Kira lived 8 months after the initial surgery and developed tumor relapse, despite 2 courses of BNCT, and we believe that the animal has probably undergone euthanasia since the owners have not responded to subsequent contact requests.

### 3.4. Case 4

**Capa**—a dog, female, 10 years old.

*Diagnosis*: Volumetric mass in the nasal cavity.

*Complaints, anamnesis*: Volumetric mass in the nasal cavity and nasal discharge.

*Examination findings:* Head and thorax CT—destructive, diffuse lesion of the nasal cavity bilaterally with lesions of the labyrinths of the grid bone, as well as subcutaneous tissue in the dorsal surface of the nasal bone on the right and frontal sinuses bilaterally. Signs of lysis of bone and cartilage structures forming nasal passages with lysis of nasal bone on the right. No metastatic lesions in the thoracic cavity were detected.

Tumor size before irradiation: 9.4 × 4.2 × 5 cm.

*Post-radiation follow-up and dynamics:* Stable satisfactory condition during the first week after radiation, with appetite, weight, and behavior remaining unchanged. Visual reduction in tumor size observed by owner. Two weeks after irradiation, the owners noted swelling in the nasal mucosa and para-orbital tissue on the left side along with decreased appetite. One month after BNCT, the appetite returned, the animal was active, and did not lose weight according to the owners. The size of the tumor had not changed and CBC was unremarkable. Two months after irradiation, a CT scan of the head was performed: the size of the tumor was 11.1 × 4.8 × 2.5 cm. The soft tissue component from the subcutaneous tissue in the area of the dorsal nasal bone decreased in dynamics. After 3.5 months, tumor growth was noted, activity and appetite decreased, discharge from the right eye appeared, nasal passage discharge resumed, and the condition was deemed to be of medium severity. Due to the progression of the tumor process and worsening of the condition, the owners decided to euthanize the dog, which occurred 4 months after BNCT.

**Figure 4:**
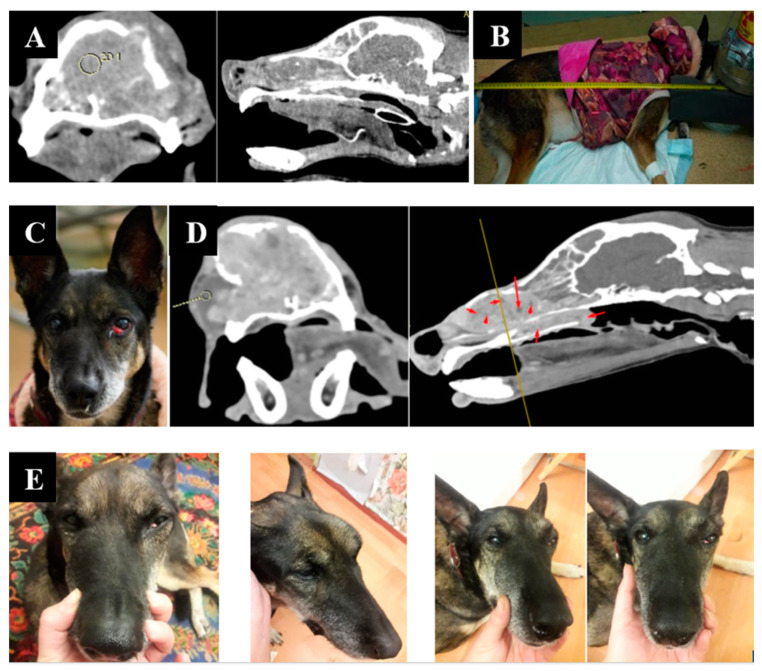
A dog named “Capa”. (**A**)—CT of the tumor before irradiation. (**B**)—positioning of the animal during the irradiation session. (**C**)—type of tumor before irradiation. (**D**)—Tumor reduction according to CT data after irradiation. (**E**)—tumor dynamics 1, 2, and 3 weeks after irradiation.

Animal appearance, irradiation positioning, and imaging results are shown in Figure 4.

### 3.5. Case 5

**Sabrina**—a female cat, age unknown.

*Diagnosis*: Tumor of the left nasal passage.

*Complaints, anamnesis:* Hemorrhagic nasal discharge.

*Examination:* Diagnostic rhinoscopy and sampling for histological study were carried out.

*Histological report:* Lymphoma, large cell, high degree of malignancy.

*Post-radiation follow-up and dynamics:* During the first week after BNCT, there was a decrease of rales, discharge from the nasal passage, and reduced appetite. At 6 weeks after treatment, the condition was stable and, while the appetite recovered, serous discharge from the left nasal passage remained. After 3 months, tumor growth resumed. The owners decided to euthanize the animal 5 months after BNCT.

**Figure 5:**
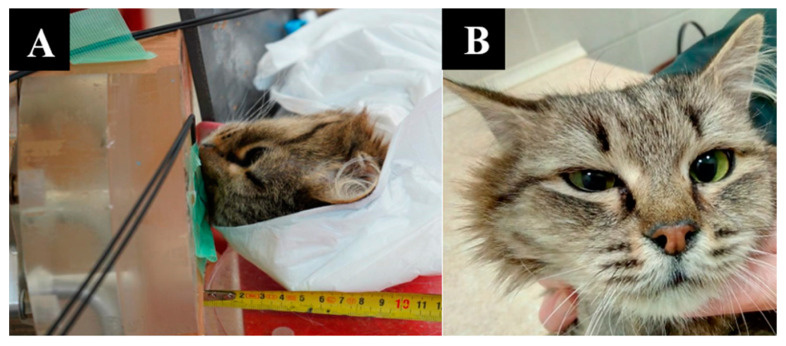
A cat named “Sabrina”. (**A**)—positioning of the animal during the irradiation session. (**B**)—The state of the animal one week after irradiation.

Irradiation positioning and animal appearance are shown in Figure 5.

### 3.6. Case 6

**Pushok**—a male cat, 8 years old.

*Diagnosis:* Tumor of the soft tissues of the nose and upper lip.

*Complaints, anamnesis:* Presence of a mass.

*Examination data:* Histological biopsy of the mass was carried out. Head CT: soft tissue mass of the lip and nose on the right side with accumulation of contrast agent; regional lymph nodes were not enlarged; no metastases were found.

*Histological report:* Squamous cell carcinoma.

*Post-radiation follow-up and dynamics:* A 70% reduction in the volume of the mass was observed visually at 1 week after irradiation. From the 6th week after radiation, renewal of tumor growth was observed. General condition was satisfactory.

**Figure 6:**
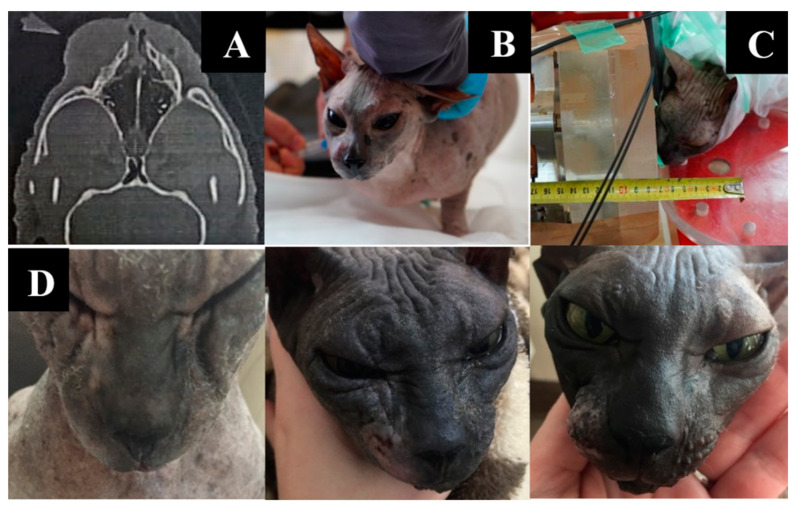
A cat named “Pushok”. (**A**)—Tumor on CT scan before irradiation. (**B**)—preparation of the animal for irradiation. (**C**)—positioning of the animal during the irradiation session. (**D**)—dynamics of changes in the size of the formation one, two, and six weeks after irradiation.

Animal appearance, irradiation positioning, and imaging results are shown in Figure 6.

Pushok developed a tumor relapse 2 months after BNCT. The owner has not responded to subsequent contact requests.

### 3.7. Case 7

**Seledka**—a female cat, 4 years old.

*Diagnosis*: Soft tissue tumor of the left femur.

*Complaints*, *anamnesis*: Impaired motor and support ability of the hind left limb, impaired limb positioning, worsened appetite.

*CT findings:* According to the CT scan, there was a soft tissue mass (in the sacro-tail ventral lateral muscle area on the left, sternum muscle on the left, and superficial and middle sternum muscles on the left) of heterogeneous density. Predominantly marginal accumulation of contrast. Polycystic kidney disease. Nephrolithiasis in the left kidney. No metastatic lesions of the thoracic and abdominal cavity were revealed.

*Histological report:* Malignant mesenchymal tumor.

*Tumor size before irradiation:* 4.1 × 2.6 × 1.8 cm.

*Post irradiation follow-up and dynamics:* At 2 weeks after irradiation, the mass was reduced by 30% (according to tomography data), pain decreased, and the appetite was restored. During 5 months, the hosts noted improvement of appetite, restoration of ability to stand, and restoration of limb function.

**Figure 7:**
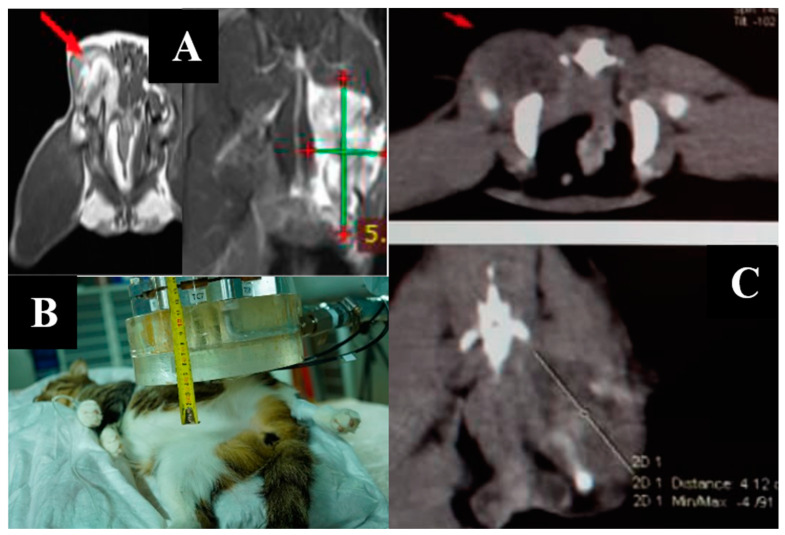
A cat named “Seledka”. (**A**)—MRI of soft tissues before irradiation. (**B**)—positioning of the animal during the irradiation session. (**C**)—CT of soft tissues three weeks after irradiation.

Imaging results and irradiation positioning are shown in Figure 7.

Seledka is alive as of 7 January 2022. Her condition is satisfactory but she has developed renal failure and has been diagnosed with pathological fracture of the femur with mild pain syndrome. The animal has normal limb placement, but is limping. A repeat session of BNCT is planned.

### 3.8. Case 8

**Fenya**—a female cat, 13 years old.

*Diagnosis*: Tumor of trapezius and rhomboid muscles.

*Complaints, anamnesis*: Impaired motor and support ability of the hind left limb, impaired limb positioning, worsened appetite.

According to CT scan of the thoracic and abdominal cavities, there was a mass in the projection of the trapezius and rhomboid muscles, mainly on the right side at the level of Th1-Th4, without invasion into the vertebral column and thoracic cavity. No metastatic lesions in the thoracic cavity were detected. Surgical removal of the tumor was performed. BNCT was performed 4 weeks after surgical treatment in the area of surgical intervention.

*Histological conclusion:* Fibrosarcoma.

*Tumor size before irradiation*: 1.0 × 2.6 × 3.9 cm.

*Post irradiation follow-up and dynamics:* The cat was monitored for 3 months after BNCT in a satisfactory condition with no signs of recurrence.

**Figure 8:**
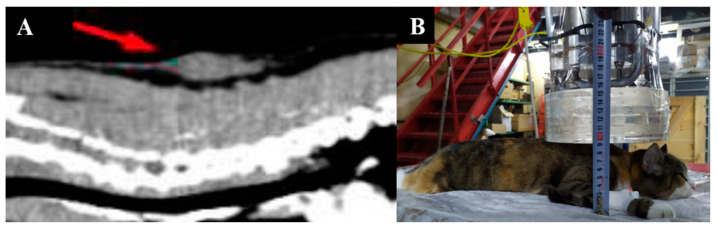
A cat named “Fenya”. (**A**)—CT of soft tissues before irradiation. (**B**)—positioning of the animal during the irradiation session.

Imaging results and irradiation positioning are shown in Figure 8.

Fenya developed tumor recurrence and underwent surgical tumor removal in the beginning of December 2021. A second BNCT session is planned.

### 3.9. Case 9

**Lucky**—a male cat, 5 years old.

*Diagnosis*: Tumor in the hyoid area.

*Complaints, anamnesis*: Presence of a tumor, worsening of appetite.

According to CT scans, the animal had a soft tissue mass in the hyoid region with indistinct borders that heterogeneously accumulated contrast substance. Hypertrophy of the soft palate was at 0.7 cm. No metastatic lesions in the chest and abdomen were revealed.

*Tumor size before irradiation*: 2.0 × 2.1 × 2.0 cm.

*Post-radiation follow-up and dynamics:* Within the first few of post-radiation follow-up, the general condition was satisfactory and improvement of appetite was noted.

**Figure 9:**
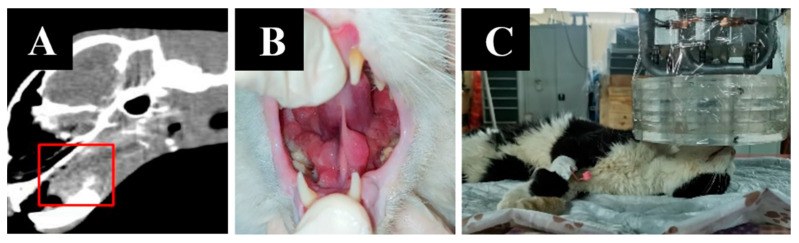
A cat named “Lucky”. (**A**)—CT of soft tissues before irradiation. (**B**)—the appearance of the tumor. (**C**)—positioning of the animal during the irradiation session.

Imaging results, animal appearance and irradiation positioning are shown in Figure 9.

## 4. Discussion

Preclinical evaluation of BNCT included the use of dogs (as animals larger than rodents) to determine the accumulation of boron compounds before treatment and evaluate the safety and efficacy of the therapy based on nuclear reactors. Takeuchi at al. (1985) performed such a study on five male dogs with naturally occurring osteosarcomas where boron accumulation after intravenous injection of BSH (50 mg of ^10^B/kg of body weight) was studied in four animals while one animal was treated with BNCT 12 h after BSH injection with a 4-h irradiation [91]. In three out of five dogs, including the irradiated animal, boron concentration in the tumor was over 30 µg/g and the authors confirmed clinical and radiological improvements in the treated animal [92].

In 1992, Kraft et al. studied biodistribution of boron in 30 dogs with spontaneous intracranial tumors after intravenous BSH (55 mg of natural boron/kg of body weight) infusion. They showed that the highest and feasible BNCT boron concentration in tumor tissue was observed at the first assessable time point of 2 h after infusion (35.9 ± 4.6 µg/g), that a high tumor/normal cell boron concentration ratio was achieved, and a subsequent linear decrease to the insufficient level of 7.0 ± 1.1 µg/g was seen at 12 h (indicating optimal time intervals for neutron irradiation) [93]. In another publication, the authors reported higher mean boron accumulation in extracranial compared to intracranial spontaneous tumors in dogs with maximum levels at 2 h after BSH infusions of the same dose as their previous report. They then described variations in tumor-to-blood and peritumor boron concentrations, suggesting further pharmacokinetic studies to evaluate whether intracranial surgeries can influence BSH pharmacokinetics [94].

To assess boron accumulation, side effects of BSH injection, and tolerability of neutron irradiation/BNCT, Gavin et al. (1994) irradiated purpose-bred laboratory dogs, including 14 dogs with epithermal neutron irradiation but without boron injection, 35 dogs irradiated after 50–100 ^10^B mg/kg BSH injection, and 4 dogs used as sham-irradiated controls [95]. The authors described tolerable doses and side effects of neutron irradiation with and without boron, including skin depigmentation, epilation and moist desquamation, skin and brain necrosis, and changes on magnetic resonance imaging [95].

In a later reactor-based study, Mitin at al. (2009) compared gadolinium neutron capture therapy (GdNCT) and BNCT in 42 dogs (33 with spontaneous oral cavity melanoma and 9 with osteosarcoma of the extremities), describing side effects of neutron irradiation, gadolinium-based, and boron-based therapies, and concluded that BNCT led to lower tissue damage than GdNCT and was more effective in superficial tumors (melanomas). GdNCT, on the other hand, showed therapeutic efficacy in canine osteosarcoma due to the benefit of secondary irradiation of these tumors [96].

In a recent study by Schwint et al. (2020), the authors performed RA-6 reactor-based BNCT in five dogs with incurable head and neck cancers (terminal condition) but without any other therapeutic options and showed both tumor response and clinical benefit with extension of animal survival time along with mild or moderate, reversible, BNCT-associated toxicity [52].

In our study, we treated dogs and cats with spontaneous tumors with different localizations at an experimental, accelerator-based BNCT facility. At the time of enrollment, all animals were incurable with a presumed acceptable quality of life of up to 3 months. One animal (Shon the pug) had severe somatic status before irradiation caused by breathing difficulties against a background of a large tumor in the nasal passages.

There were no animals in our study that had previously received chemotherapy. However, since, in some animals, chemotherapy intolerance has been reported, we would have included these animals as BNCT was the only available radiation therapy.

We used BSH as a boron compound due to its availability and our previous small animal experience [74,75]. Based on literature data, we estimated the tumor-to-healthy tissue ratio of boron as 3:1 while the tumor/blood ratio was assumed to be 1:1 [52,93]. Boron concentrations in blood (and in the tumor, assuming a tumor/blood ratio as 1:1) before and after irradiation were higher (Table 3) than the initially considered minimum boron concentration in tumors (30 ppm). We started irradiation ≤ 10 min after the end of the BSH infusion and continued for an average of two hours. This interval was chosen due to the fact that, based on our previous studies on BSH biodistribution in mice, tumor boron concentration decreases rapidly to sub-therapeutic levels over the 2-h period after infusion of a standard dose of 100 mg/kg body weight [74,75]. Kraft et al. (1992) showed the maximum boron concentration at the first 2-h timepoint after BSH infusion with further linear decrease to sub-therapeutic concentrations [93]. Therefore, we believe that the early start of irradiation in our cases was justified according to these similar reports.

After BNCT, all other animals, except for Shon, showed positive dynamics in the form of tumor volume reduction and clinical improvement (evidenced in appetite and lessened symptoms). Among the adverse reactions were mild and reversible dermatitis, color changes, and hair loss in the area of irradiation. In one case of double irradiation, we observed post-irradiation necrosis of the skin at the irradiation site. Nausea and casual vomiting were observed in all animals during and after BSH infusion. No cases of mucositis have been observed or reported by the animal owners or veterinarians. As for Shon, enrolled as a last-resort attempt, the animal was already in terminal condition with breathing difficulties even before the irradiation (with little tolerance to treatment) and died a few hours after the BNCT session from postintubation mucosal swelling but without developing mucositis.

The results of imaging studies 1–3 months after BNCT revealed either no changes or decreases in tumor volume, indicative of beneficial effect or at least a halting of tumor progression for all animals. In most animals, post-BNCT survival varied, based on both treatment results and owner discretion, but generally ranged from 3 to 12 months. As of this moment, three animals are confirmed to be alive and, in Case 1, positive dynamics with decreased size of lung metastases and no visible tumor tissue in the nasal area were reported. In some cases, animal owners did not respond to subsequent contact requests and, thus, detailed descriptions of those cases remain incomplete.

BNCT prolonged expected survival by an average of 3 months and markedly improved quality of life. As a rule, as early as 14 days after BNCT, a positive response to treatment contributed to a notable improvement in the animals’ general condition and quality of life. The absence of tumor size changes after irradiation and recurrence in most cases can be explained by the fact that the tumors were large and, as such, the deep-tumor irradiation dose was likely insufficient while the low selectivity of BSH as a delivery agent for boron to tumor cells might have also played a role. Possible improvements for future trials could include several sessions of BSH, the use of combinations of boron-containing drugs, and the combined use of adjunct therapies (including radiation therapy).

The disadvantages of the study include a small sample size, different histological characteristics of the tumors, the use of different animal species, and the absence of a reference tomographic control for tumor size. Future experiments should also add a tumor-bearing control group to the study, as exposing healthy animals to possible harm to study the effects of AB-BNCT on different tissues (such as in previous reactor-based studies by other researchers) is not ethically suitable. Such control groups would include pets with spontaneous tumors treated with standard therapy.

At the present time, BNCT is a last resort for treating animals with spontaneous malignant tumors for which no other therapies are feasible. This work is hereby presented as a pilot project to introduce new veterinary technology for treating malignant tumors in Russian pets since animal radiation therapy in our country is completely absent. Additionally, as an intermediate step between murine studies and human trials, larger-animal studies such as this allow for testing of current BNCT technology while providing a framework for both future veterinary and clinical studies.

## 5. Conclusions

This study is a continuation of a series of BNCT preclinical biological studies. Its distinctive feature is the use of an accelerator neutron source, which can be installed in clinics, that features safety advantages over nuclear reactors [52,96]. The data obtained indicate a partial tumor response when performing BNCT with an accelerator neutron source, both as a monotherapy method and in combination with surgical treatment and chemotherapy. The availability of a safe and reliable source of epithermal neutrons for BNCT, in light of reactor-based safety restrictions, makes global testing feasible and encourages synergistic development of animal and human BNCT therapy for both veterinary and clinical applications.

## Figures and Tables

**Table 1:** Data on gender, species, age, localization and type of tumors in animals participating in the study.

N	Nickname	Species	Age	Tumor Localization	Histological Diagnosis
1	Daya	dog	12	soft tissue of the facial skull	osteosarcoma
2	Perchik	cat	4	mammary gland, frontal bone	carcinoma
3	Kira	cat	6	soft tissue of the back	fibrosarcoma
4	Kapa	dog	10	nasal cavity	-
5	Sabrina	cat	unknown	nasal meatus (passage)	lymphoma
6	Pushok	cat	8	nose and upper lip area soft tissues	squamous cell carcinoma
7	Seledka	cat	4	soft tissue of the thigh	mesenchymal tumor
8	Fenya	cat	13	trapezius and rhomboid muscles (after tumor removal)	fibrosarcoma
9	Lucky	cat	5	hyoid area	-
10	Shon	dog	11	upper jaw, hard palate	fibrosarcoma

**Table 2:** Parameters used for irradiation dose calculation.

Proton Beam Energy, MeV	Dose Rates, Gy/mC			
	Thermal Neutrons	Fast Neutrons	Boron-Related for 1 ppm	Photons
	Tumor			
2.05	3.94 × 10^−5^	1.41 × 10^−5^	1.91 × 10^−5^	3.98 × 10^−4^
2.10	5.07 × 10^−5^	2.58 × 10^−5^	2.45 × 10^−5^	5.08 × 10^−4^
	Surrounding healthy tissues (brain)			
2.05	5.22 × 10^−6^	9.42 × 10^−7^	2.52 × 10^−6^	1.20 × 10^−4^
2.10	6.75 × 10^−6^	1.86 × 10^−6^	3.26 × 10^−6^	1.52 × 10^−4^
	Skin			
2.05	4.46 × 10^−5^	3.96 × 10^−5^	2.17 × 10^−5^	3.74 × 10^−4^
2.10	5.61 × 10^−5^	6.84 × 10^−5^	2.73 × 10^−5^	4.67 × 10^−4^

The calculations were carried out for the following parameters: irradiated area at a distance of 1 cm from the target, tumor located at a depth of 2 to 6 cm with a volume of 6 × 3 × 3 cm.

**Table 3:** Data on the concentration of boron in the blood and parameters of irradiation in animals participating in the study.

N	Nickname	Species	Boron Concentration in Blood, µg/g		Irradiation Parameters					
			Before Irradiation	After Irradiation	Energy (MeV)	Current (mA)	Current Integral (mAh)	Neutron Fluence (×10^14^ n/cm^2^)	Tumor Dose (Gy-eq)	Skin Dose (Gy-eq)
1	Daya	dog	149 128	119 104	2.1 2.05	2 2.6	3.3 4	1.95 1.79	42.1 39.5	14.5 13.3
2	Perchik	cat	138	98	2.05	2.6	4	1.79	39.5	13.3
3	Kira	cat	99 124	74 70	2.05 2.05	2.6 2.6	4 2.7	1.79 1.21	39.5 26.7	13.3 9
4	Kapa	dog	99	65	2.1	2.1	4.4	2.60	56.2	19.3
5	Sabrina	cat	60	32	2.05	1.8	4.4	1.97	39.5	13.3
6	Pushok	cat	80	50	2.05	1.8	4.4	1.97	39.5	13.3
7	Seledka	cat	120	59	2.1	2.1	3.9	2.31	49.8	17.1
8	Fenya	cat	167	92	2.05	1.8	4	1.79	39.5	13.3
9	Lucky	cat	75	44	2.05	1.8	4	1.79	39.5	13.3
10	Shon	dog	109	44	2.1	2	4	2.37	51	17.6

The irradiation doses are total absorbed doses. Neutron fluence represents total number of neutrons generated in each session. Neutron fluence calculations based on Lee et al. (1999) [90]. The actual neutron fluence corresponds to the calculated one [82].

## Data Availability

The data presented in this study are available upon request from the corresponding author.
